# Insights into Inhalation Drug Disposition: The Roles of Pulmonary Drug-Metabolizing Enzymes and Transporters

**DOI:** 10.3390/ijms25094671

**Published:** 2024-04-25

**Authors:** Liuhan Dong, Xiaomei Zhuang

**Affiliations:** Beijing Institute of Pharmacology and Toxicology, Beijing 100850, China; donglh_cy@163.com

**Keywords:** pulmonary pharmacokinetics, drug transporters, metabolizing enzymes, inhalation administration

## Abstract

The past five decades have witnessed remarkable advancements in the field of inhaled medicines targeting the lungs for respiratory disease treatment. As a non-invasive drug delivery route, inhalation therapy offers numerous benefits to respiratory patients, including rapid and targeted exposure at specific sites, quick onset of action, bypassing first-pass metabolism, and beyond. Understanding the characteristics of pulmonary drug transporters and metabolizing enzymes is crucial for comprehending efficient drug exposure and clearance processes within the lungs. These processes are intricately linked to both local and systemic pharmacokinetics and pharmacodynamics of drugs. This review aims to provide a comprehensive overview of the literature on lung transporters and metabolizing enzymes while exploring their roles in exogenous and endogenous substance disposition. Additionally, we identify and discuss the principal challenges in this area of research, providing a foundation for future investigations aimed at optimizing inhaled drug administration. Moving forward, it is imperative that future research endeavors to focus on refining and validating in vitro and ex vivo models to more accurately mimic the human respiratory system. Such advancements will enhance our understanding of drug processing in different pathological states and facilitate the discovery of novel approaches for investigating lung-specific drug transporters and metabolizing enzymes. This deeper insight will be crucial in developing more effective and targeted therapies for respiratory diseases, ultimately leading to improved patient outcomes.

## 1. Introduction

**Figure 1:**
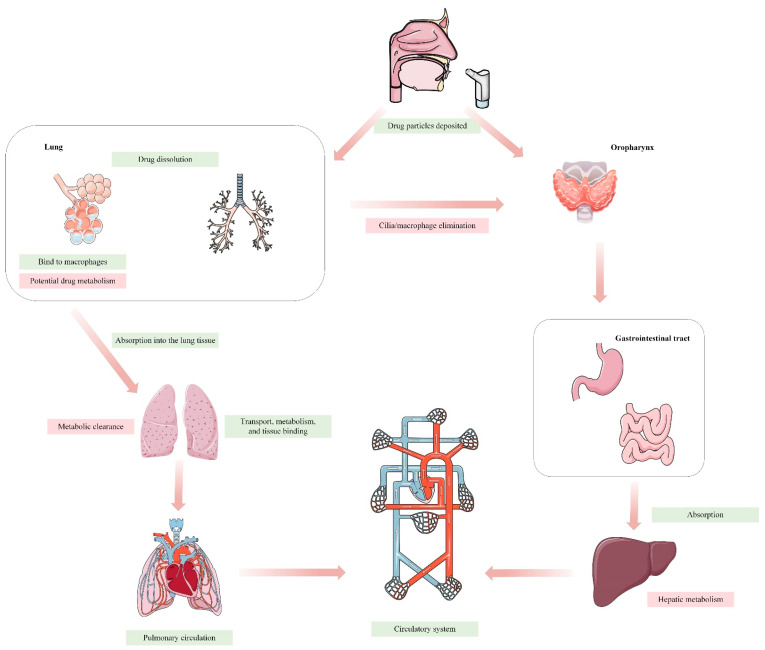
Summary of the lung-specific PK processes for inhaled drugs.

In recent decades, there has been a significant advancement in contemporary lung delivery techniques for the treatment of respiratory ailments globally. The lung, a vital organ for gas exchange, also serves as a non-invasive route for drug delivery. It directly influences the local elimination of inhaled medications and systemic elimination of pharmaceuticals absorbed into the lung through blood circulation [1]. Inhaled formulations are widely favored for preventing and treating acute and chronic respiratory disorders, attributed to the lung’s low enzyme activity and effective drug absorption, bypassing the first-pass effect. Inhalation has emerged as a promising non-invasive approach for the delivery of biological macromolecules and proteins, thereby enhancing patient adherence. This is particularly evident in the context of inhaled insulin therapy for diabetes [2,3]. However, the complexity and heterogeneity of the respiratory tract also pose challenges in targeted administration of inhaled medications. Unlike oral ingestion, administering medications via the lung involves unique processes of absorption, distribution, metabolism, and elimination (Figure 1). Medications must navigate physiological processes, including mucociliary clearance and macrophage-mediated phagocytosis, before reaching the alveoli. Among the many factors that affect drug delivery [4], particle size is the main factor affecting the deposition performance of the lung. The size of the particles has a substantial impact on where and how the inhaled particles are distributed in the lungs. Particles ranging in size from 5 to 10 μm are primarily deposited in the oropharynx. Particles measuring 2–5 μm in size are primarily deposited in the lungs and bronchi, with 50–60% of them being deposited in the alveoli. Particles with a size smaller than 1 μm are unable to reach the outer regions of the lungs and will be exhaled while breathing, resulting in no therapeutic benefits [5]. The distribution of drug deposition in the lungs varies across pulmonary regions, which complicates an accurate assessment of absorption. Natural pulmonary defense mechanisms further complicate drug deposition assessment by effectively removing inhaled drug particles [6,7]. Substances introduced into the lungs, including drugs, undergo various physiological processes like transport, metabolism, tissue binding, and systemic circulation entry [5]. Understanding the pharmacokinetic (PK) processes of pulmonary medicines is inherently complex due to these factors. Furthermore, changes in the physiological state of the respiratory system during disease conditions introduce additional complexities. Illnesses that affect the respiratory tract can alter the expression and function of transporters and enzymes, potentially impacting the efficacy and safety of inhaled medications. Understanding the interplay between respiratory health and drug disposition is crucial for optimizing treatment plans and managing potential drug interactions or adverse effects.

The significance of membrane transporters and metabolic enzymes involved in oral medications is well recognized. They play critical roles in drug disposition, intracellular concentration, and biological activity, impacting therapeutic effectiveness and patient safety [8]. Extensive research has been conducted in the gut, liver, kidney, and brain to study their involvement in absorption and elimination processes. A growing body of research has demonstrated that the lungs contain a variety of membrane transporters as well. Among these, certain members of the solute carrier family (SLCs) are primarily responsible for the uptake of substances, while ATP-binding cassette (ABC) transporters actively facilitate the secretion of compounds from the cell [9,10]. Drug metabolism enzymes, including cytochrome P450 (CYPs), glutathione S-transferase (GST), sulfotransferase (SULT), epoxide hydrolase (EPX), and glucuronosyltransferase (UGT), which are often expressed and studied in the liver, are also found in extrahepatic organs such as the lung [11,12]. Nevertheless, the current understanding of drug transporters and metabolizing enzymes in lung tissue both locally and systemically and their impact on drug pharmacokinetics and pharmacodynamics remains limited due to insufficient knowledge.

This review aims to comprehensively outline the available literature on the expression of transporters and metabolizing enzymes in the lung, shedding light on their impact and research techniques concerning the absorption, disposition, metabolism, and excretion of exogenous and endogenous chemicals. Furthermore, it will address advancements made in research in this field.

## 2. Expression and Function of Transporters in the Lungs

**Figure 2:**
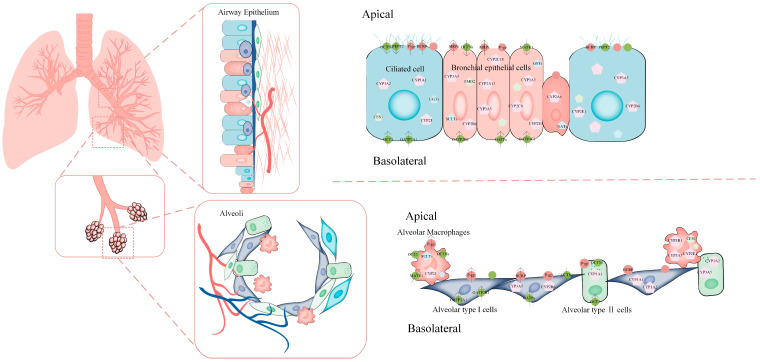
Pulmonary drug transporters and metabolic enzymes. (Green circles indicate uptake transporters and red circles indicate efflux transporters; pentagons indicate metabolic enzymes).

Transporters in the lung, like in other human tissues, encompass ABC transporters and the SLC superfamily, suggesting that drug absorption in the lung involves active mechanisms beyond passive diffusion (Table 1 & Figure 2). However, much of the existing evidence is derived from organoid models cultured in vitro or collected ex vivo. These studies establish the in situ expression of drug transporters in lung tissue and demonstrate transporter-related actions in cultured cells.

### 2.1. ABC Transporters

ABC transporters are a significant group of transmembrane proteins that function as ATP-dependent efflux pumps, expelling a wide array of chemically distinct compounds from the cytoplasm to the extracellular environment. Current research has extensively focused on transporters such as P-glycoprotein (P-gp), breast cancer resistance protein (BCRP), and multidrug resistance-related protein (MRP), with limited studies on vault proteins (MVPs), ABCAs, and other less common transporters.

**P-glycoprotein (P-gp)**, also known as MDR1 or ABCB1, is primarily expressed in the small intestine, liver, proximal renal tubules, and the apical membrane of the blood–brain barrier. In rodents and humans, P-gp is predominantly present in bronchial airway epithelial cells, alveolar macrophages, and various cell types in peripheral lung tissue [13,14]. Furthermore, no statistically significant distinction was detected between individuals diagnosed with chronic obstructive pulmonary disease (COPD) and those who were deemed healthy controls [15,16]. A study employed isotope dilution nanoLC-MS/MS technology to measure the expression levels of pulmonary transporters and indicated that the expression of P-gp transporters was comparatively lower than that of MRPs and BCRP. These studies suggest that P-gp may play a relatively minor role in pulmonary efflux, with lower expression compared to MRPs and BCRP [17].

**Breast cancer resistance protein (BCRP) transporter**, encoded by the ABCG2 gene, is notably expressed in various tissues including the placenta, gastrointestinal system, brain, liver, breast, and cancer cells. Currently, there is a limited body of research pertaining to pulmonary BCRP transporters, and existing studies exhibit significant heterogeneity [13,14,18]. In primary cultured human whole lung tissue and bronchial epithelial cells, the expression level of BCRP detected by LC-MS/MS was the second highest among all ABC transporters. However, in alveolar and tracheal epithelial cells, it was below detection limits [19]. Nickel et al. [20] found that BCRP is differently expressed in AT2 and AT1-like cells with lower abundance and activity in the latter ones, due to its expression being decreased during transdifferentiation from AT2 to AT1-like phenotype. In primary cells, the protein was found in the nucleus in addition to the cell membrane, and it might play a transcriptional role in distal lung epithelium. The mRNA expression of the BCRP transporter was found to be reduced in both the central and peripheral airways of individuals who were healthy as well as those diagnosed with COPD. Protein expression of BCRP in human lungs appears to be modest [21].

**Multidrug Resistance-Associated Protein (MRP)** is implicated in multidrug resistance and transports a range of complexes across cellular membranes. The MRP protein family encompasses nine members, namely MRP1–9. Notably, MRP1, MRP6, and MRP7 exhibit substantial expression in the lung and airway. However, the expression levels of MRP2, MRP8, and MRP9 were found to be significantly low or absent in previous studies [22,23]. MRP1 is the predominant ABC transporter found in the human lung, primarily localized to the basolateral membrane of bronchial and alveolar epithelial cells. Regional variations in protein expression of MRP1 have been observed, with higher levels detected in the bronchi and alveoli compared to the trachea. Levels of MRP3, MRP5, and MRP8 were found to be comparatively elevated in females as compared to males [22,24]. Several researchers have provided evidence supporting a substantial correlation between MRP transporters and the incidence and severity of respiratory disorders. This finding implies that MRP transporters might have a heightened significance in individuals afflicted with respiratory conditions [25]. The recent discovery that tobacco smoke and inhaled medications have an impact on the expression and function of MRP1 in human distal lung epithelial cells in a laboratory setting implies that this transporter may be expressed differently in individuals who smoke or patients with certain medical conditions [26].

**ABCAs** facilitate lipid transport in the respiratory system and have implications in various diseases. The mRNA expression of ABCA transporters is significant in both central and peripheral airways, varying among epithelial cells in successive airway generations [27]. The regulation of ABCA1 and ABCA13 is impacted by exposure to cigarette smoke, with ABCA13 expression showing differential expression in patients diagnosed with COPD and asthma. The expression of ABCA-3 is significantly elevated in type-II alveolar cells, and deleterious mutations in this gene have been identified as a causative factor for severe respiratory disease in newborns and children [18].

**Vault proteins (MVPs)**, including LRP-56, LMR-5, and MVP-37, are also expressed in the pulmonary system. LRP, a type of vault protein, is detected in lung epithelial cells and shows elevated expression in lung cancer tissues, suggesting its potential as a biomarker and therapeutic target for lung cancer [28].

The current knowledge of ABC transporter expression in the human lung remains a subject of debate within the academic community, particularly regarding their levels of expression, location, and functionality in healthy lung tissues. Conflicting findings also exist concerning their overexpression in lung cancer and its contribution to multidrug resistance and unfavorable prognosis for patients. Further research is warranted to elucidate their precise roles under physiological and pathological states of the lung.

### 2.2. SLC Transporters

Solute carriers (SLCs) constitute a diverse class of transmembrane transporters, exceeding 300 members, primarily located on cellular membranes [29]. These transporters play a key role in enhancing the transportation of various substrates across biomembranes, including the uptake of small molecules across biomembranes. Notable efforts have been focused on organic cation transporters (OCTs/OCTNs) and organic anion transporting polypeptide (OATPs), encompassing a significant portion of the existing knowledge. Additionally, uptake transporters like oligopeptide transporters (PEPTs), mammal multidrug and toxin extrusion proteins (MATEs), and serotonin transporter (SERT) have recognized physiological functions within the lungs.

#### 2.2.1. Lung Organic Cation Transporters (OCTs/OCTNs)

In the lungs, organic cation transporters comprise five primary isoforms: OCT1, OCT2, OCT3, OCTN1, and OCTN2, all classified under the SLC22A transporter family [30]. Notably, research on the expression of this transporter family has been more extensive compared to others. For instance, internal data from GlaxoSmithKline (GSK) suggest that considerable genomic expression data of these transporters in human lung tissue should be collected [17].

- **OCTN1 and OCT3** are highly present in the lung, with the highest mRNA expression intensity observed in bronchi and reduced levels in peripheral lung tissues [17,19,31,32]. They are found in various lung cell types, including bronchial and alveolar epithelial cells, bronchiolar wall epithelial cells, alveolar macrophages, and type-II cells.
- **OCTN2** displays a similar expression pattern to OCTN1 and is present in the bronchial area and bronchiolar wall epithelial cells, among others.
- **OCT1 and OCT2** do not have significant expression in the lungs, although limited expression of OCT2 has been observed in some individuals. For example, a limited expression of OCT2 in the lungs of a few Caucasian and Spanish subjects was reported [17].

#### 2.2.2. Organic Anion Transporting Polypeptides (OATPs)

The OATP family plays a significant role in transporting and eliminating both endogenous and exogenous organic anions. The expression levels of OATP transporters were observed to be the most prominent in the lung, with subsequent expression levels observed for PEPT2 and ABC transporters BCRP and P-gp [19]. Among transporters studied, OATP2A1 and OATP2B1 are more prevalent in the lung compared to others. OATP2A1 protein expression is notably prominent, but smoking-induced inflammation may reduce its concentration [17,19,32]. Studies [28] have shown that the expression level of OATPs in the human lung is OATP2A1 > 2B1 > 4C1 > 3A1 > 4A1, and OATP1A2 was not expressed. However, Bleasby et al. [33] found that the order was OATP2A1 > 3A1 > 2B1 > 4A1 > 4C1 > 1A2.

#### 2.2.3. Mammalian Multidrug and Toxin Extrusion Proteins (MATEs)

MATE1 mRNA expression levels are notably higher in human peripheral lung tissue compared to bronchial tissue [31]. MATE1 protein is present in bronchial and bronchiolar epithelial cells, lymphocytes (especially T cells), and alveolar macrophages in the peripheral lung. The detection of MATE2 expression was unsuccessful [31].

#### 2.2.4. Oligopeptide Transporters (PEPTs) and Serotonin Transporter (SERT)

PEPT2D mRNA expression is significantly higher in both central and peripheral airways, whereas Fallon et al. [17] reported that the levels of PEPT2 protein expression were relatively low compared to OATP2A1. SERT mRNA exhibits prominent expression in both the central and peripheral airways, playing a pivotal role in pulmonary vascular remodeling/adaptation during neonatal development [32].

Understanding the expression and functions of these transporters and lung physiology is vital for comprehending drug pharmacokinetics and their impact on lung pathology. The precise roles of these transporters in the lungs necessitate further research for elucidation.

## 3. Expression and Function of Metabolizing Enzyme in the Lungs

While the liver and intestine are paramount in drug metabolism, the lung, receiving the full cardiac output, possesses a significant metabolic capacity due to its high blood flow [1]. Numerous drug-metabolizing enzymes are present in the lung, including cytochromes P450 (CYPs), flavin-dependent monooxygenase (FMO), alcohol dehydrogenase (ADH), NAD(P)H:quinone acceptor oxidoreductase (NQO), hydrolases, glutathione-S-transferase (GST), UDP-glucuronosyl transferase (UGT), sulfotransferase (SULT), and N-acetyl transferase (NAT) enzymes [11,34] (Table 1 & Figure 1). Pulmonary P450 enzymes play a crucial role in bioconversion, deactivation of inhaled carcinogens, and detoxification of pneumotoxins. Various P450 enzymes are expressed in the lung, with specific ones like CYP1A1, CYP1B1, CYP2A6, CYP2B6, CYP2C18, CYP2E1, CYP2F1, CYP2J2, CYP2S1, and CYP3A5 being present. Additionally, CYP2S, CYP2F, and CYP4B1 are regarded to be specific to the lung [35,36,37]. These enzymes are distributed across different cell types in the lung, including alveolar cells, goblet cells, ciliated epithelial cells, and vascular epithelial cells. Non-ciliated bronchiolar epithelial cells (clara cells) and type-II lung cells may also exhibit P450 enzyme presence, albeit in relatively lower abundance. Although the functionality of P450 enzymes in the lung is weaker due to their limited content [12,38], FMO2, a specific variant of flavin monooxygenase, is notably expressed in the pulmonary system, unlike the hepatic system.

Some phase-I metabolic enzymes are specifically expressed in the respiratory system or have higher expression in the lung than other tissues, such as CYP2A13, CYP2F1, CYP3A5, CYP4B1, etc. Among these, CYP2A13 has a high level of protein expression in human bronchial and tracheal epithelial cells, while it is rarely distributed in alveolar cells [39]. However, CYP2A6, which has a high amino acid sequence similarity with CYP2A13, is not only distributed in respiratory tissues but is expressed in bronchial epithelial cells of the peripheral lung [40]. CYP2F1 is capable of bioactivating a number of pulmonary-selective toxicants, and its expression is highly tissue-selective; the highest expression is observed in the lung with little or no hepatic expression. This is due to the existence of mechanisms that govern the unique tissue-specific regulation of CYP2F1 [41]. CYP3A5 is the predominant CYP3A form in the human lung, localized by immunohistochemistry in the ciliated and mucous cells of the bronchial wall, bronchial glands, bronchiolar columnar and terminal cuboidal epithelium, type-I and type-II alveolar epithelium, vascular and capillary endothelium, and alveolar macrophages. CYP3A4 is expressed in about 20% of individuals, and considerable variation of pulmonary expression occurs in both CYP3As between individuals [42].

In addition to phase-I metabolic enzymes, the lung houses several phase-II metabolic enzymes such as UGTs and STs. Notably, human lung microsomes possess prostaglandin H synthase (PGHS), an arachidonic acid-dependent peroxidase absent from the hepatic system. The peroxidation activity of PGHS enables the activation of specific carcinogenic arylamine compounds, highlighting its distinctive role in pulmonary physiology [34,38].

Numerous studies have consistently demonstrated a general decrease in the activity of cytochrome P450 enzymes within the pulmonary system across diverse species and various in vitro models. However, the significance of other oxidoreductases with potential importance in the lung’s metabolism warrants further exploration. Recent research has focused on the impact of pulmonary metabolizing enzymes on the elimination of inhaled medications, shedding light on their intricate role in drug metabolism within the lung [43].

## 4. Contribution of Pulmonary Transporters to Drug Disposition

Historically, research on pulmonary transporters has primarily focused on the challenges posed by upregulated efflux proteins in effective pulmonary delivery. Excessive expression of ABC transporters, associated with multidrug resistance (MDR) in cancer cells, significantly diminishes drug concentration within cells [44]. Given the increased emphasis on inhaled medications [45], recent research has shifted its focus towards comprehending the impact of drug transporters on absorption and distribution within the pulmonary system.

Both SLC and ABC transporters play vital roles in transmembrane transport and barrier enhancement through substrate ejection [8,32,46,47]. The presence of biomembrane, however, presents a significant impediment to the efficient delivery of hydrophilic drugs to intracellular targets, deeper tissues, or the systemic pulmonary circulation. Despite this, limited research has explored the influence of these barriers on pulmonary drug delivery. Pulmonary efflux transporters significantly affect the efficacy and safety of inhaled medications. Modulating these transporters could enhance pharmacokinetic and pharmacodynamic properties of pulmonary-administered medicines.

The role of OCT and OCTN in facilitating the internalization of nature products and medications in lung cells has been extensively documented. These transporters handle various endogenous substances including neurotransmitters, L-carnitine, and ergothioneine. The OCT/Ns protein has been found to have a role in facilitating the transportation of xenobiotics across different biological barriers. This includes substances such as biguanides and histamine receptor antagonists, as demonstrated in previous studies [47,48,49]. Moreover, it should be noted that several pharmaceuticals employed in the treatment of respiratory ailments exhibit cationic properties under physiological pH conditions, hence making them prospective substrates for organic cation transporters/novel substrates. OCT/Ns have been found to potentially contribute to chronic lung disorders’ pathogenesis. Consequently, they present promising therapeutic targets. Studies have investigated these transporters’ role in transepithelial substance uptake into the pulmonary circulation, predominantly utilizing in vitro cell models. In an investigation conducted by Nakamura et al. [50], uptake assays were performed on human bronchial epithelial BEAS-2B cells and HEK293 cells overexpressing OCTN1/2, revealing that ipratropium and tiotropium exhibited predominant absorption through OCTN2 in bronchial epithelial cells. These findings suggest a reduced level of absorption via OCTN1, consistent with the pharmacological effects of the drug upon inhalation. Similarly, there is a limited availability of ex vivo models that can be used to supplement the findings obtained at the cellular level. Al-Jayyoussi et al. [51] examined the role of OCTs and OCTN transporters in the overall lung uptake of L-carnitine and ipratropium. They utilized an intact in vitro perfused rat lung (IPRL) model to investigate this contribution and compared the findings with active transport in vitro using three human lung cell lines and primary rat alveolar epithelial cells. The findings revealed that the contribution of OCT/OCTN transporters to pulmonary uptake of L-carnitine or ipratropium at the ex vivo level was negligible, contradicting in vitro studies which suggested their significant role in cellular substrate accumulation. These studies indicated a preferential uptake of ipratropium by OCT and L-carnitine by OCTN [47,52]. Consequently, it can be inferred that relying solely on in vitro absorption studies may pose challenges when accurately predicting in vivo absorption.

Recent advancements employ positron emission tomography (PET) methodologies to evaluate pulmonary transporters, specifically focusing on efflux transporters [53]. PET imaging, using radiotracers, aids in understanding the distribution and movement of substrates in the lungs. Studies have demonstrated the impact of P-gp and BCRP on the distribution of inhaled medications in animal models. Irene et al. [15] measured the intrapulmonary pharmacokinetics of the model P-gp substrates (R)-[^11^C] verapamil ([^11^C] VPM) and [^11^C]-N-desmethyl-loperamide ([^11^C] dLOP) using PET imaging. The findings indicate that the presence of P-gp can influence the distribution of inhaled substances that are substrates of P-gp in the lungs. A decrease in P-gp activity may lead to reduced pulmonary exposure to these substances, potentially compromising the efficacy of therapeutic interventions. This study emphasizes the potential of utilizing PET imaging with intratracheally aerosolized radiotracers to evaluate the influence of membrane transporters on the administration of drugs to the lungs, in both rodent models and potentially in human subjects. Severin et al. [54] conducted a study to investigate the intrapulmonary pharmacokinetics of the intratracheally (i.t.) aerosolized model P-gp substrate [^11^C]metoclopramide. Their study aimed to compare the pharmacokinetics in the presence and absence of P-gp activity using a comparable positron emission tomography (PET) technique. The findings indicate that the transport of [^11^C] metoclopramide across the epithelial layer is not significantly influenced by P-gp activity, or is minimally affected. Moreover, the elevated accumulation of [^11^C] metoclopramide in the lungs may be attributed to a decrease in P-gp-mediated elimination into the bloodstream when P-gp activity is absent in the endothelial cells of the capillaries. The membrane transporter P-gp is located in the apical (i.e., lumen-facing) membrane of airway epithelial cells and in the luminal (blood-facing) membrane of pulmonary capillary endothelial cells. Therefore, the interplay between lung epithelial and endothelial cell contacts may influence the transport of a substrate for P-gp. BCRP is an additional type of efflux transporter that is found in the apical membranes of lung epithelial cells, specifically towards the airway lumen. In order to assess the impact of P-gp and BCRP on the pulmonary distribution of inhaled medications, Severin et al. [14] conducted PET imaging experiments on rats. The rats were exposed to two radiotracers, namely [^11^C] erlotinib and [^11^C] tariquidar, which served as representative substances that are substrates for P-gp and BCRP. These radiotracers were administered to the rats via intratracheal aerosolization. The findings of this study indicate that there is in vivo functional activity of P-gp and BCRP in the lungs. The obtained results are consistent with the hypothesis that there exists functional redundancy between P-gp and BCRP, akin to the observed phenomenon at the blood–brain barrier which restricts pulmonary uptake of model substrates [55].

In summary, recent research elucidates the pivotal role of pulmonary transporters in facilitating drug delivery to the lungs. A comprehensive understanding of their functionality and impact on drug absorption, distribution, and efficacy is imperative for optimizing inhaled drug therapies. Targeting these transporters holds immense potential to revolutionize pulmonary drug delivery, thereby enhancing the effectiveness of therapeutic interventions.

## 5. Contribution of Pulmonary Metabolizing Enzymes to Drug Disposition

The enzymatic activity in the lungs profoundly influences the efficacy of inhaled medications, which is contingent upon the temporal and spatial distribution of substrates, ultimately governing their accessibility to enzymes. In vitro methodologies have predominantly focused on investigating the metabolism of corticosteroids and β2 agonists using human tissue. However, with the emergence of novel drug classes, there has been a shift in focus towards the development of new formulations for inhaled medications and an increasing emphasis on studying biologics and drug excipients’ metabolism [56]. Human cytochrome P450 (CYP) enzymes play a significant role in drug detoxification, cellular metabolism, and maintaining homeostasis. Approximately 80% of oxidative metabolism and around 50% of the overall clearance of market drugs can be attributed to one or more distinct P450 isoforms. In addition to their pivotal role in metabolic processes, cytochrome P450 enzymes (CYPs) possess the ability to modulate pharmacological action, safety, bioavailability, and resistance within metabolic organs and specific drug target sites [1,11,34]. A recent review conducted by Enlo-Scott et al. has elucidated the importance of comprehending and predicting the pulmonary metabolism of inhaled pharmaceuticals to optimize their clinical efficacy [34].

Ciclesonide, an inhaled medication, has undergone extensive research regarding its pulmonary metabolism. Various in vitro models, including precision-cut lung slices, the A549 cell line resembling type-II alveolar cells, primary bronchial epithelial cells, and primary nasal epithelial cells, have demonstrated processes involving hydrolysis and fatty acid conjugation associated with ciclesonide [57,58,59,60,61,62]. In lung tissue samples, ciclesonide undergoes an initial conversion to the active metabolite desisobutyryl-ciclesonide, followed by subsequent transformation into fatty acid conjugates. This reversible synthesis of fatty acid conjugates has been identified as a significant metabolic pathway in human lung slices for ciclesonide [62], which has consequently established its well-established utilization as an inhaled prodrug [63]. Fluticasone propionate, an inhaled corticosteroid primarily used for asthma and COPD [64], has been studied in in vitro tests to elucidate its pulmonary metabolism. The sole metabolite observed in humans is the 17β-carboxylic acid derivative of fluticasone propionate, believed to be generated via the CYP3A4 metabolic pathway [65]. However, Pearce et al. [66] investigated this phenomenon in human pulmonary microsomes and recombinant CYP 3A4, 3A5, and 3A7 enzymes, revealing a lack of metabolite formation. These findings were corroborated by Navel et al., who also observed the absence of detectable metabolites using the human lung precision-cut technique [67]. Wakayama et al. [68] developed an in silico prediction system, indicating that fluticasone propionate is anticipated to undergo metabolism mediated by CYP3A4, with potential contributions from other CYP enzymes.

Roflumilast, a prodrug with long-acting bronchodilator properties used for COPD, is under consideration for inhalation administration to mitigate potential gastrointestinal side effects [69]. Its active metabolite, roflumilast N-oxide, is primarily generated by hepatic CYP1A2 and CYP3A4 enzymes. The presence of pulmonary enzymes CYP1A2 and CYP3A5 suggests the potential for activation when directly administered to the lungs, albeit possibly at a reduced rate. Roflumilast, another drug, has demonstrated efficacy in mouse models of asthma following inhalation, which motivates further exploration into appropriate pulmonary formulations [70].

## 6. DDI Associated with Pulmonary Transporters/Metabolizing Enzymes

Both the ICH and the FDA have issued or updated guidelines that address drug–drug interactions (DDIs) [71,72]. These guidelines provide a framework for researching interactions between drugs and enzymes or transporters, essential for the development of new therapeutics. Naturally, inhaled medications are also subject to these guidelines. However, to date, establishing an in vitro/in vivo correlation for pulmonary drug DDIs presents unique challenges due to the distinct absorption, distribution, metabolism, and excretion (ADME) processes at play in respiratory drug delivery, as well as the specialized characteristics of pulmonary metabolic enzymes and transporters.

Itraconazole, known for its potent inhibitory effect on cytochrome P450 3A4 (CYP3A4), is implicated in DDIs. The administration of PUR1900, a dry powder formulation of itraconazole for oral inhalation, has been shown to achieve substantial drug concentrations in the lungs while keeping systemic levels low. In a study by Mackenzie et al. [73], a physiologically based pharmacokinetic (PBPK) model was built, with midazolam as the “victim drug,” to assess the potential for DDIs with inhaled PUR1900. Both the basal static and mechanistic static models in the study indicated a higher probability of DDI occurring after inhaling PUR1900. However, clinical data from a phase-1 study of PUR1900, when reconciled with PBPK model simulations, indicated that at a maximum dose of 40 mg, the geometric mean ratios for midazolam’s C_max_ and AUC were 1.14 and 1.26, respectively. This suggests a minimal risk of DDIs associated with PUR1900 inhalation. The low systemic exposure to itraconazole through PUR1900 significantly reduces the potential for CYP3A4 inhibition, thereby diminishing concerns regarding DDIs from oral inhalation of the drug.

In vitro evidence obtained from the low-dose inhalation of the phosphatidylinositol 3-kinase δ inhibitor, nemiralisib, indicates its role as both a substrate and a strong metabolism-dependent inhibitor of CYP3A4 and P-gp substrates. Aarti et al. [74] demonstrate that nemiralisib is unlikely to cause any clinically significant DDIs by combining computer modeling, in vitro testing, and clinical trials. They also found that a variety of commonly co-administered medications can be safely included in clinical study designs for patients. Although inhaled drugs are generally expected to have limited systemic exposure, adherence to regulatory guidance, including the tiered approach for perpetrator interactions, is essential. A comprehensive evaluation of potential DDIs for inhaled compounds is recommended, considering therapeutic area-specific issues, in vitro perpetrator liabilities, and victim investigations.

## 7. Refining the Measurement and Prediction of Transport and Biotransformation Processes within the Pulmonary System

A significant challenge in studying drug transporters and metabolizing enzymes in the lungs is the limited availability of tools to assess local drug elimination [47,75]. Despite these challenges, there are several methods relying on either ex vivo animal models or in vitro human/animal models [76,77]. For instance, animal models translating in vitro to in vivo (clinical) settings offer substantial benefits in investigating metabolic pathways. Our research team has been investigating the ADME processes of various medications throughout the entire animal body, with a specific focus on drug delivery through the pulmonary route and its impact on lung-specific disposition. However, due to the intricate nature of in vivo drug disposition, elucidating the influence of individual factors on drug disposition poses significant challenges in terms of both complexity and cost. Cell culture retains transporters and metabolic enzymes provide a practical platform for characterization of substrate specificity, drug interactions, and kinetic aspects. The utilization of ex vivo lungs allows the examination of drug transport and metabolism within a preserved lung architecture, thereby facilitating the integration of the administration and clearance of an inhaled drug [78,79]. In the context of advancing computer technology, leveraging in silico methods for prediction has become possible, relying on ex vivo or in vitro preclinical animal data [80,81,82]. The following sections introduce a variety of alternative methodologies of animal experimentation.

### 7.1. In Vitro Methods

In cases where the drug target is intracellular, traditional macroscopic pharmacokinetic investigations may not accurately reflect the drug concentration at a specific target site. This disparity can impede the correlation between systemic medication concentration and its therapeutic target. Therefore, conducting cellular pharmacokinetic studies on lung inhalation formulations is essential to understand the mechanisms and extent of their penetration into target cells. This understanding is crucial for advancing lung inhalation preparations.

In the context of localized drug administration, lung inhalation agents directly affect respiratory cells, and the effectiveness of drug uptake by these cells is closely linked to therapeutic effects. Cellular uptake investigations enable the visual assessment of the uptake efficacy of lung-inhaled formulations compared to a pure solution. While extensive research has been conducted on the gene expression profiles of enzymes and transporters in lungs, there has been relatively less emphasis on investigating the challenges associated with optimizing drug metabolism via inhalation, such as exploring enzyme activity [83].

When selecting cell models, it is important to note that certain cell lines, such as A549 (resembling alveolar epithelial type-II cells) and BEAS2B (a bronchial epithelial cell line), offer advantages over primary tissue models. These benefits include reduced genetic variation, continuous culturing capabilities, lower financial costs, and ease of implementation in high-throughput assays. However, it is important to acknowledge that cell lines might experience modifications in protein expression and, in certain instances, demonstrate reduced activity compared to original cells (Table 2). Consequently, the true range and magnitude of drug disposition in the lungs may be underestimated [84].

There is a higher probability that immortalized cell lines derived from non-neoplastic sources will demonstrate a characteristic phenotype in terms of enzyme expression. For instance, studies have demonstrated that the immortalized bronchial cell line BEAS-2B exhibits a higher resemblance to the gene expression observed in primary bronchial tissues, as well as cell lines derived from cancer. Similarly, research has indicated that the immortalized alveolar type-I cell line TT1 displays greater sensitivity compared to the cancer-derived A549 alveolar type-II cell line [85]. Additionally, it has been proposed that culturing lung epithelial cells under air–liquid interface (ALI) conditions promotes a more differentiated phenotype, as demonstrated by sustained elevation of surfactant protein levels or the reemergence of cilia [86]. Moreover, ALI culturing may influence the expression of transporters [86,87], imposing more rigorous operational criteria for cell culture protocols. Although the culture conditions and quality control standards were intricate, we successfully established a calu-3 cell air–liquid interface (ALI) culture model to assess drug permeability across the bronchial epithelial cell barrier. The obtained results demonstrated concordance with the drug uptake outcomes observed in A549 cells.

In summary, cell culture models provide a convenient and efficient approach for investigating the molecular-level mechanisms underlying drug disposal. Absorption, transport, metabolism, and toxicity studies can be conducted in vitro, without the complexities of organ systems or preclinical animals. However, it is imperative to acknowledge the limitations inherent in these models, such as the absence of clearance systems and their tendency to primarily represent homozygous cell types. These constraints should be taken into consideration when analyzing data obtained from in vitro experiments [88].

In recent years, multiple cell co-culture models mimicking lung cells have been rapidly developed. These co-culture models, simulating lung cells, are being investigated to address the limitations associated with using a single cell type in drug transport and metabolic research within the context of lung cells [89,90,91,92,93]. Kletting et al. [90] successfully developed a lentiviral-immortalized human alveolar epithelial cell line referred to as hAELVi, which, when cultured on polyester semipermeable membranes, closely resembles alveolar type-I cells in terms of functionality and morphology. Subsequent co-cultivation of the THP-1 human cell line with these cells confirmed that the resulting co-culture effectively functioned as a barrier [91,94]. Brookes et al. [93] constructed an air–liquid interface (ALI) model comprising alveolar epithelium consisting of type-I (hAELVi) and type-II (NCI-H441) cells. They assessed gene expression associated with barrier function and differentiation, critical processes in this context. The co-culture model demonstrated a persistent barrier similar to the barrier observed in primary human alveolar epithelial-like cells, as revealed by the study. Moreover, this co-culture model exhibited expression of surfactant protein C and displayed an appropriate expression profile of claudins and aquaporins for the distal lung. A recent study [95] introduced novel findings on “Arlo”, a newly developed monoclonal cell line derived from hAELVi. The Arlo cell line was created using single cell printing, and its functional properties were thoroughly examined. This study highlights the potential of Arlo as a fundamental component in constructing human alveolar epithelial models and in developing more intricate in vitro models in the future. This presents an additional innovative and compelling framework for characterizing substance distribution throughout the lungs.

Subcellular fractions offer numerous advantages in investigating lung metabolic enzymes and are widely used as an in vitro approach for studying drug metabolism [1]. Despite existing studies on the lung S9 fraction, a composite preparation comprising both cellular microsomal and cytosolic components [96], isolated microsomes remain the predominant subcellular fraction employed in drug metabolism research. Isolated microsomes contain significant drug metabolizing enzymes, including CYP and UGTs, offering distinct advantages in terms of cost-effectiveness and ease of utilization. Commercially available lung microsomes can be procured or isolated from various sources, such as rabbit lungs, rat lungs, human lungs, and lung cell lines like the A549 human lung cancer cell line. It is important to note that microsomes prepared from different sources may exhibit variations in protein content [97]. Cryopreservation and storage of cell cultures offer advantages over tissue samples, primarily in terms of convenience and suitability for high-throughput kinetic studies. Cell cultures can be easily cryopreserved and stored, ensuring their long-term preservation. Moreover, they exhibit reduced biological variability across experiments, rendering them more consistent in scientific investigations. In contrast to whole-cell models that require substrates to traverse intracellular pathways, subcellular components tend to overestimate metabolic clearance due to the facile creation of substrate-enzyme complexes in the absence of structural barriers. For instance, salbutamol sulfation demonstrates that the metabolic clearance of salbutamol is significantly lower in intact bronchial epithelial cells compared to human lung cytoplasm [98].

The use of isolated lung cells has demonstrated its efficacy as a model system for investigating the cellular regulation of the CYP pathway and the involvement of CYP enzymes in lung detoxification and metabolic activation. Despite the considerable variation among animals, it has been observed that clara cells are characterized by a profuse presence of smooth endoplasmic reticulum. Consequently, these locations have the potential to serve as focal points for CYP-dependent activity. Various techniques for isolating alveolar type-II cells and clara cells across diverse animals have been documented [99,100,101,102]. Typically, investigations have primarily focused on the expression and activity of CYP within airway epithelial cell lines, specifically secondary cultures. These cell lines include normal human bronchial epithelial (NHBE) cells, Calu-3, 16HBE14o-, BEAS-2B, and the type-II alveolar cell line A549. UDP-glucuronosyltransferases (UGTs), representative of phase-II metabolic enzymes, were identified within NHBE, Calu-3, and BEAS-2B cells [103,104]. Several publications have proposed that the activity of CYP enzymes is slightly reduced, and the expression pattern is slightly modified in normal human bronchial epithelial (NHBE) cells compared to in vivo. The A549 cell line has been extensively characterized for its conjugative enzyme activity, while its oxidase activity is suboptimal and its overall metabolic capacity is believed to be constrained. The Calu-3 cell line exhibits polarization when grown as a monolayer, generating cilia and mucus. This has led researchers to consider it appropriate for experiments on absorption and medication metabolism. The 16HBE14o cell line originates from the conversion of bronchial epithelial cells, and it displays comparable characteristics to those of the Calu-3 cell line [105]. Numerous researchers have investigated the expression and activity of enzymes in various cell models [37,104,106,107,108,109]. However, there remains a lack of clarity regarding whether the metabolic activities in specific cell types adequately reflect those in the corresponding lung tissue.

### 7.2. Ex Vivo Methods

Ex vivo lung models offer a valuable platform for investigating the efficacy and therapeutic impact of inhaled medications in both healthy and diseased pulmonary systems. Utilizing the ex vivo lung model preserves the 3D structure of the lung and its native microenvironment, allowing for interactions between cells and the extracellular matrix. Consequently, ex vivo drug studies enable specific examination of the impact of the in vivo lung environment on various aspects, including drug transport kinetics, cytotoxicity, and therapeutic efficacy [110].

Using accurately dissected lung sections as an in vitro model is highly valuable due to its ability to preserve the 3D lung structure, shape, and cellular diversity. This approach provides a more accurate representation of in vivo conditions, demonstrating superior fidelity in terms of relevant protein expression and function when compared to alternative in vitro models. However, primary cells are frequently employed due to challenges of tissue acquisition and experimental complexity [111,112].

Precisely cut lung sections (PCLSs) refer to lung tissue sections from donors that are thinly sliced (100–300 µm) and preserved in a medium. This method aims to maintain the lung’s functional physiology and cellular interactions while conserving its morphological and structural variety [113]. It has emerged as a powerful research tool, offering a more complex mechanistic understanding of drug interactions with the multicellular and multidimensional properties of the lung. Yildiz et al. [38] utilized PCLSs to compare lung metabolism between rats and humans, revealing that both species had relatively low CYP activity compared to the liver, with phase-II activity being more prominent. The study also observed distinct differences in rat lung CYP activity compared to human lung CYP activity. Additionally, the study assessed the stability of CYP enzymes and their associated enzyme systems in lung section cultures. Previous research has shown a decline in heterologous metabolic enzymes in rat lung sections over time in cultures. The PCLS model system offers an alternative to conventional tissue culture by cultivating lung sections from healthy and diseased tissues across various species, showing promise for reducing reliance on animal experimentation in studying lung diseases. To enhance the existing model, various methods for high-throughput processing and culture can be implemented, along with improved drug delivery strategies [113,114].

Isolated perfused lungs (IPLs) present a distinctive opportunity to assess the aerodynamic performance and bio-pharmacokinetics of inhaled drugs [110]. The platform closely emulates the physiological and biological characteristics of lungs in preclinical species, facilitating investigations into drug aerodynamics and bio-pharmacokinetic properties. Understanding drug penetration through the pulmonary epithelium, drug absorption velocities, and therapeutic effectiveness are crucial aspects evaluated using IPL [115,116,117]. Eriksson et al. [117] utilized drug transport data acquired from inhaled chemicals in the single-pass, isolated perfused rat lung model to enhance the comprehension of pulmonary epithelial transport for their investigational compound. The model effectively described the pulmonary drug transport profiles and the impact of formulations with varying characteristics on the overall absorption rate in the pulmonary system. Sjogren et al. [118] examined the dissolution of four inhaled drugs with low solubility when delivered as either suspension or dry powder in an established ex vivo isolated perfused lung model. The in vitro dissolution data were close to the ex vivo lung dissolution data, suggesting the potential to enhance the in vitro lung model. IPL provides a physiologically relevant framework for evaluating novel inhalation therapies targeting the tracheal, bronchial, and alveolar regions. However, it is noteworthy that a significant proportion of studies utilizing IPL primarily focus on healthy animal lungs. It is crucial to accurately simulate the mechanical, structural, and physiological characteristics of diseased lungs [119] in order to obtain valuable data on aerodynamics and bio-pharmacokinetics during the preclinical evaluation of inhaled therapies.

### 7.3. In Silico Methods

**Figure 3:**
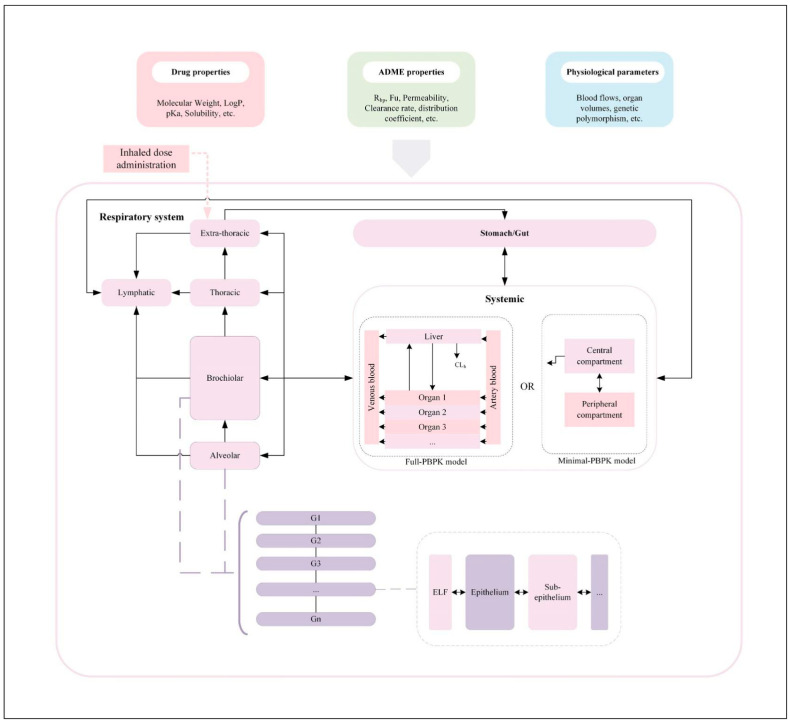
Typical PBPK model for inhalation formulations.

While there are many in silico techniques for predicting drug disposition, only a limited subset has been used to evaluate lung-specific absorption, disposition, metabolism, and excretion (ADME). A fundamental challenge in computer-based methodologies is accurately predicting the ADME characteristics of drug prototypes (both parent and metabolites). These predictions, such as solubility, permeability, or metabolic stability based solely on a structure–activity relationship (SAR), often rely on training datasets from established compounds. This restricts the ability to project outcomes for unique or innovative chemical structures [80,120,121,122]. To overcome this challenge, it is imperative to employ software technologies that capitalize on existing advancements in inhalation deposition prediction, metabolic prediction, and respiratory bioavailability pharmacokinetic prediction to accurately forecast and elucidate the metabolic destiny of inhaled pharmaceuticals [122,123]. The rapidity of computerized techniques, compared to in vitro procedures, allows for accelerated initial assessment of inhaled drug metabolism, aiding in the design of in vitro human or in vivo animal investigations. The integration of physiologically based pharmacokinetic (PBPK) modeling (Figure 3) and microarray analysis is seen as a promising and influential approach in pharmaceutical research, offering a potential alternative to animal experimentation for advancing novel drug candidates. A review presents recent advancements in microarray tissue technology and PBPK modeling, encompassing model construction, parameter estimation, and validation strategies [124]. Modeling PBPK based on microarray tissue is anticipated to become an integral part of new drug development, personalized medicine, and related fields.

Miller et al. [125] developed a PBPK model to predict the concentrations of nemilarix in plasma and tissues following inhalation delivery. The model, constructed using the GastroPlus^®^ software platform (version 9.7, https://www.simulations-plus.com/software/gastroplus/), integrated a comprehensive mechanistic representation of pulmonary absorption, systemic distribution, and oral absorption subsequent to nemilarix inhalation. Model validation was facilitated by clinical data obtained through intravenous, oral, and inhaled delivery, enabling accurate evaluation of pulmonary drug absorption using observational data. To substantiate the efficacy of computer simulation as a valuable method for the development and assessment of inhaled products, Tang et al. [81] chose to utilize indacaterol, a long-acting β2 adrenergic agonist widely recognized as a primary treatment option for patients with COPD. The PBPK model developed in the study exhibited robust stability and demonstrated broad applicability. Throughout the modeling process, it became evident that the size of drug particles significantly impacted pharmacokinetics (PK). Statistical analysis demonstrated that maintaining bioequivalence relative to the baseline (5 μm) was achievable when the particle size of the active pharmaceutical ingredient (API) varied within the range of 3.5 to 6.5 μm. In practical application, precise data on medication characteristics, including the particle size of APIs and carriers, can be used to construct models that align with observed pharmacokinetic (PK) outcomes. Consequently, it is widely acknowledged that PBPK modeling holds significant potential as a valuable tool for advancing inhaled products while simultaneously reducing costs and time associated with animal experimentation and clinical trials. However, it is crucial to emphasize the necessity of sufficient model validation to ensure the accuracy of model application.

The advantages and disadvantages of each method are summarized in Table 3.

## 8. Conclusions

The field of drug transporters and metabolism in the lungs is rapidly advancing due to technological advancements, innovative research methodologies, and an increasing recognition of the significance of pulmonary drug disposition in therapeutic outcomes. However, there are substantial challenges that must be addressed to bridge the gap between research and clinical practice. The focus of future investigations should be on refining and validating in vitro and ex vivo models, enhancing our understanding of drug disposition in different disease states, and developing novel approaches to study lung-specific drug transporters and metabolizing enzymes. Collaboration among researchers, clinicians, pharmacologists, and computational scientists will be crucial for addressing these challenges and propelling the field forward. By understanding how drugs are transported, metabolized, and eliminated in the lungs, we can optimize treatment strategies for respiratory diseases while improving drug efficacy and minimizing adverse effects. Ultimately, this knowledge will contribute to the development of safer and more effective inhalation medications that benefit patients.

## Figures and Tables

**Table 1:** Expression of major drug transporters and metabolic enzymes in human lung tissue.

	Gene	mRNA Level	Protein Expression
**Transporter**			
**ABC Transporter**			
MDR1/P-gp	*ABCB1*	+++	++
MRP1	*ABCC1*	++	++
MRP2	*ABCC2*	+/−	+/−
MRP3	*ABCC3*	+	+
MRP4	*ABCC4*	+/−	+
MRP5	*ABCC5*	+	+
MRP6	*ABCC6*	++	+
MRP7	*ABCC10*	++	+/−
MRP8	*ABCC11*	−	−
MRP9	*ABCC12*	−	−
BCRP	*ABCG2*	++	++
**SLC transporter**			
OCT1	*SLC22A1*	+/−	+/−
OCT2	*SLC22A2*	−	−
OCT3	*SLC22A3*	++	++
OCTN1	*SLC22A4*	++/+	++
OCTN2	*SLC22A5*	+	++/+
OATP1A2	*SLCO1A2*	+/−	+/−
OATP1B3	*SLCO1B3*	+/−	ND
OATP2A1	*SLCO2A1*	+	++
OATP2B1	*SLCO2B1*	++/+	++
OATP3A1	*SLCO3A1*	+	+
OATP4A1	*SLCO4A1*	++	+
OATP4C1	*SLCO4C1*	+	+
PEPT1	*SCL15A1*	−	−
PEPT2	*SCL15A2*	++	+
MATE1	*SLC47A1*	+	+
SERT	*SLC6A4*	++	+
**Enzyme**			
**Phase I**			
CYP1A1	*CYP1A1*	+++/−	++
CYP1A2	*CYP1A2*	+/−	+
CYP1B1	*CYP1B1*	++/+	+
CYP2A6	*CYP2A6*	++/+	++
CYP2A13 *	*CYP2A13*	+/+++	ND
CYP2B6/7	*CYP2B6/7*	+++	+
CYP2C8	*CYP2C8*	+/−	+
CYP2C9	*CYP2C9*	+/−	ND
CYP2C18	*CYP2C18*	+	+
CYP2C19	*CYP2C19*	+/−	ND
CYP2D6	*CYP2D6*	+/−	ND
CYP2E1	*CYP2E1*	+++/++/+	+
CYP2F1 *	*CYP2F1*	+++/++	ND
CYP2J2	*CYP2J2*	+	+
CYP2R1	*CYP2R1*	+	ND
CYP2S1	*CYP2S1*	+	ND
CYP2W1	*CYP2W1*	++	ND
CYP3A4	*CYP3A4*	+/−	ND
CYP3A5 *	*CYP3A5*	+++/++	++
CYP3A7	*CYP3A7*	+/−	ND
CYP3A43	*CYP3A43*	+/−	ND
CYP4B1 *	*CYP4B1*	+	ND
CES1	*CES1*	++	++
FMO2	*FMO*	+	+
**Phase II**			
GSTs	*/*	++	++
NATs	*/*	++	+
SULTs	*/*	+	+
UGTs	*/*	+	−

**Note:** ABC (ATP-binding cassette), P-gp (P-glycoprotein), MRP (multidrug resistance-related protein), BCRP (breast cancer resistance protein), SLC (solute carrier), OCT (organic cation transporter), OATP (organic anion transporting polypeptide), PEPT (oligopeptide transporter), MATE (mammal multidrug and toxin extrusion protein), SERT (serotonin transporter), CYP (cytochrome P450), CES (carboxyl esterase), FMO (flavin-dependent monooxygenase), GST (glutathione-S-transferase), NAT (N-acetyl transferase), SULT (sulfotransferase), and UGT (UDP-glucuronosyl transferase). +++: high level; ++: middle level; +: low level; −: very low level or not found; ND: no data. *: Expressed selectively in the lungs compared to other organs.

**Table 2:** Cell lines commonly used in in vitro models of the lung.

Cell Line	Transporter Expression	Enzyme Expression	Characteristic
**Calu-3**	**P-gp**, MRP1, MRP2, **BCRP** *, OCT1, OCT3, OCTN1, OCTN2	ND	➢ Used as proximal lung model. ➢ Capable of forming a barrier. ➢ Represents bronchial epithelium.
**BEAS-2B**	MRP1, **BCRP**, OCT1, OCT2, OCT3, OCTN1, OCTN2, OATP1A2	CYP1B1, CYP2R1, CYP2S1, CYP2U1, CYP4A11, CYP4A22, CYP4B1, CYP4F11, CYP4F12, CYP4V2, CYP4X1, CYP4Z1, CYP5A11, CYP7B1, CYP8A1, CYP20A1, CYP21A2, CYP27A1, CYP27B1, CYP27C1, CYP39A1, CYP51A1, CES2, CES3, FMO2, FMO3, FMO4, FMO5, ADH1B, AKRs, EPHX1, EPHX2, NQO1, NQO2, GSTA4, GSTP1, SULT1E1, UGT3A2	➢ Long-lasting characteristics. ➢ Capable of terminal differentiation. ➢ Ideal for assessing substances affecting differentiation and tumor formation.
**16HBE14o-**	ND	CYP1B1, CYP2E1, CYP2J2, CYP2S1, CYP2U1, CYP4F3, CYP4V2, CYP7B1, CYP8A1, CYP20A1, CYP24A1, CYP26B1, CYP27B1, CYP27C1, CYP39A1, CES2, CES3, FMO4, FMO5, ADH5, ADHFE1, AKRs, EPHX1, EPHX2, NQO1, NQO2, GSTA4, GSTP1, NAT1, NAT5, SULT1A3, SULT1A4, SULT2B1	➢ Displays characteristics of bronchial epithelial cells. ➢ Exhibits cobblestone morphology and cytokeratin expression. ➢ Forms tight junctions and supports ion transport. ➢ Utilized as a benchmark in cystic fibrosis research.
**NCI-H292**	**P-gp**, MRP1, MRP2, **BCRP**, OCT1, OCT2, OCT3, OCTN1, OCTN2	CYP1A1, CYP1B1, CYP2A7, CYP2A13, **CYP2B6**, **CYP2D6**, CYP2E1, CYP2J1, CYP2R1, CYP2S1, CYP2U1, CYP2W1, **CYP3A5**, CYP4B1, CYP4F11, CYP4F12, CYP4V2, CYP4X1, CYP7B1, CYP8A1, CYP20A1, CYP21A2, CYP24A1, CYP26A1, CYP26B1, CYP27B1, CYP39A1, CYP51A1, CES2, CES3, FMO4, FMO5, ADH5, ADHFE1, AKRs, EPHX1, EPHX2, NQO1, NQO2, GSTA4, GSTP1, NAT1, NAT5, SULT1A2, SULT1A2, SULT1A4, SULT1E1, SULT2B1, UGT3A2	➢ Positive immunoreactivity for keratin and vimentin. ➢ Sensitive to HBV. ➢ Used in research on HBV and other virus-induced pneumonia and lung cancer.
**NCI-H441**	**P-gp**, MRP1, MRP2, MRP3, **BCRP**, OCT1, OCT2, OCT3, OCTN1, OCTN2, PEPT2	ND	➢ Mimics type-II epithelial cells. ➢ Forms tight junctions. ➢ Produces type-II markers, including surfactant proteins. ➢ Clara cell granules in the endoplasmic reticulum.
**A549**	**P-gp**, MRP1, MRP2, MRP3, **BCRP**, OCT1, OCT3, OCTN1, OAT4, PEPT1,	CYP1A1, CYP1B1, CYP2A7, **CYP2C9**, CYP2C18, CYP2J2, CYP2R1, CYP2S1, CYP2U1, **CYP3A5**, CYP4F3, CYP4F11, CYP4F12, CYP4V2, CYP5A11, CYP7B1, CYP20A1, CYP24A1, CYP26A1, CYP26B1, CYP27B1, CYP27C1, CYP39A1, CYP51A1, CES1, CES2, CES3, FMO4, FMO5, ADH5, AKRs, EPHX1, EPHX2, NQO1, NQO2, GSTA1, GSTA2, GSTA4, GSTP1, NAT1, NAT5, SULT1A3, SULT1A4, SULT1C2, SULT2B1, UGT1A1, UGT1A6, UGT1A7, UGT1A8, UGT1A9, UGT2A3, UGT2B10, UGT2B11, UGT2B15, UGT2B28, UGT2B4, UGT2B7	➢ Used to model the distal lung. ➢ Lack of tight junction proteins. ➢ Inability to form barrier.

**Note:** ND: no data. *: The data has wide variability among studies.

**Table 3:** Advantages and disadvantages of different methods studying pulmonary drug disposition.

Model	Advantages	Disadvantages
**In vitro**	➢ Ethical acceptability. ➢ More cost-effective and logistically simpler.	
Primary cells (bronchial or alveolar)	➢ Protein expression closely mirrors in vivo conditions. ➢ Allows for diverse or monoculture cellular experimental setups. ➢ Offers increased throughput compared to ex vivo models.	➢ High cost. ➢ Time limitations affect culture. ➢ Preservation and protein expression dynamics. ➢ Genetic variance among donors.
Cell lines	➢ More readily accessible. ➢ Diminished genetic variety compared to the basic models. ➢ Possibility to incorporate co-cultures. ➢ Suitable for high-throughput testing. ➢ Unnecessary inclusion of cofactors. ➢ Easier to maintain.	➢ Lacks replication of normal cell physiology and morphology. ➢ Displays altered protein expression compared to primary cells and natural lung diversity.
Lung cell homogenate	➢ High-throughput detection. ➢ Easily acquired from primary tissues or cell lines. ➢ Enzyme-induced metabolism is not hindered by cellular uptake requirements.	➢ Activity may be lower in whole cells or subcellular fractions. ➢ Overestimation of clearance due to increased substrate/enzyme access. ➢ Tissue-derived samples might show lower activity. ➢ Cofactors might be required for optimal activity (e.g., NADPH, UDPGA, PAPS).
S9 fraction	➢ Commonly used for in vitro metabolic studies. ➢ High-throughput detection. ➢ Covers phase-I and -II enzymes.	➢ Cofactors may be needed (e.g., NADPH, UDPGA, PAPS). ➢ Clearance may be overestimated due to increased substrate/enzyme access. ➢ Lower activity possible in whole tissue samples.
Microsomes	➢ Well-suited for high-throughput detection. ➢ Focuses on ER-bound enzymes like CYPs, UGTs. ➢ Higher metabolic rates due to enzyme abundance. ➢ Includes recombinases for specific CYP isoforms.	➢ Excludes pathways like EST, SULT, NAT, GST. ➢ May overestimate clearance due to increased substrate/enzyme access. ➢ NADPH cofactor is required.
Cell cytosol	➢ High-throughput detection.	➢ Ignores microsomal metabolism. ➢ May require cofactors (e.g., NADPH, UDPGA, PAPS).
**Ex vivo**	➢ Preserves lung’s 3D structure and environment. ➢ Allows for detailed analysis of drug transport, toxicity, and efficacy.	
Precision-cut lung slices	➢ Easy to acquisition of rodent lung pieces. ➢ 3D structures mimicking natural lung with enzyme expression. ➢ Human lung tissue can be utilized.	➢ The cost of human lung pieces is high. ➢ Experiment length limited by tissue life. ➢ Variability in donor genetics. ➢ Differences in protein expression between species. ➢ Rat metabolic rate may overestimate metabolite yields.
Isolated perfused lungs	➢ Mimics in vivo conditions closely. ➢ Aerosol administration for inhalation mimicry. ➢ Preserves tissue and cell structural integrity. ➢ Allows for concurrent pharmacokinetic data collection.	➢ Primarily dependent on animal models, such as rats. ➢ The high metabolic rate may lead to an overestimation of rat metabolite production. ➢ Interspecies differences. ➢ Low-throughput detection. ➢ Using solely for identification may miss some metabolites.
**In vivo**	➢ Accepted by regulatory bodies. ➢ Enhances realism in pharmacokinetic translation.	➢ Constraints with unknown metabolites in new compounds and low-dose medications. ➢ Physiological differences between species. ➢ High costs and long study durations. ➢ Ethical concerns.
**In silico**	➢ More flexibility, lower costs. ➢ Clarify key in vivo disposal mechanisms. ➢ Reduce animal testing burden following the 3Rs.	➢ Limited models for the evaluation of pulmonary disposition. ➢ Commonly disseminated through web services, with a majority of them being offered as commercial software solutions. ➢ Limited by detection system reliability and data extraction.
